# *ApaI* Polymorphism in the Vitamin D Receptor Gene Decreases the Risk of Perianal Fistulas in Crohn’s Disease

**DOI:** 10.3390/nu16203485

**Published:** 2024-10-14

**Authors:** Laura Gisbert-Ferrándiz, Jorge Llau, Dolores Ortiz-Masia, Jesús Cosín-Roger, Dulce Carolina Macias-Ceja, Joaquín Hinojosa, Sara Calatayud, Maria Dolores Barrachina

**Affiliations:** 1 Departamento de Farmacología and CIBERehd, Facultad de Medicina, Universidad de Valencia, Av. Blasco Ibáñez, 15, 46010 Valencia, Spain; laura.gisbert@uv.es (L.G.-F.); jorgellau1@gmail.com (J.L.); jesus.cosin@uv.es (J.C.-R.); macias.dcc@gmail.com (D.C.M.-C.); sara.calatayud@uv.es (S.C.); 2 Departamento de Medicina and CIBERehd, Facultad de Medicina, Universidad de Valencia, Av. Blasco Ibáñez, 15, 46010 Valencia, Spain; m.dolores.ortiz@uv.es; 3 Servicio de Gastroenterología, Hospital de Manises, 46940 Valencia, Spain; jhinojosad@gmail.com

**Keywords:** vitamin D receptor, single-nucleotide polymorphisms, *ApaI*, *TaqI*, *BsmI*, *FokI*, perianal fistula, Crohn’s disease, penetrating behavior, fibroblasts

## Abstract

Background: Vitamin D, through the activation of its receptor (VDR), plays an immunomodulatory role in the gastrointestinal tract. Single-nucleotide polymorphisms (SNPs) in the VDR gene have been associated with Crohn’s disease (CD) risk, and patients carrying the *TaqI* polymorphism in this gene run a higher risk of developing a penetrating behavior. Aims: We analyzed the association of *BsmI*, *ApaI*, *TaqI*, and *FokI* SNPs in the *VDR* gene with the clinical characteristics of CD. Methods: Four polymorphisms identified in the *VDR* gene (*BsmI*, *FokI*, *ApaI*, and *TaqI*) were genotyped in blood samples from CD patients (n = 115) by using PCR-RFLP. The disease’s location and behavior and the presence of perianal fistulas were collected from each patient. Intestinal fibroblasts from ileal resections of CD patients (n = 10) were genotyped, and the expression of fibrotic and inflammatory markers was analyzed by RT-PCR. Results: The data reveal no association between any of the polymorphisms and CD risk. A strong linkage disequilibrium was detected between *TaqI* and both *ApaI* and *BsmI*, which in turn were strongly associated. Homozygosis or heterozygosis for the a allele of the *ApaI* SNP or b allele of the *BsmI* SNP was significantly associated with a lower risk of a penetrating behavior, while the aa genotype was associated with a lower risk of perianal fistulas. Fibroblasts carrying the aa genotype expressed lower levels of fibrotic and inflammatory markers. Conclusion: The aa genotype of the *ApaI* SNP in the *VDR* gene is associated with a lower risk of perianal fistulas in CD and a reduced expression of fibrotic and inflammatory markers in intestinal fibroblasts.

## 1. Introduction

Crohn’s disease is a chronic inflammatory disorder of the gastrointestinal (GI) tract associated with transmural inflammation affecting the entire thickness of the bowel wall, and it is characterized by periods of relapse and clinical remission. Although some doubts arise [1], the Montreal Classification is the most widely accepted and integrates disease location, disease behavior, and the presence of perianal disease. The colon and ileum constitute the main organs affected, even though CD can affect any part of the GI tract. Disease behavior is classified into three phenotypes: inflammatory (B1), stenotic (B2), and penetrating (B3). Additionally, perianal disease must be noted separately, as it is not necessarily associated with fistulizing disease [2,3,4]. While disease location tends to remain relatively static, both disease behavior and perianal disease can vary over time. In fact, patients with an inflammatory phenotype at diagnosis are very likely to develop either fistulizing or stricturing complications within a few years.

Both the etiopathogenesis of CD and the reasons why some patients evolve toward more complicated behaviors are unknown. Genetic studies have identified susceptibility genes for CD [5], among which, the human *VDR* gene, located on chromosome 12q13.11 is situated within one of the candidate regions for inflammatory bowel disease (IBD) susceptibility [6,7]. This nuclear receptor mediates most of the biological functions of VD and it has been associated with an immunomodulatory effect in the gastrointestinal tract [6]. Four common single-nucleotide polymorphisms (SNPs) recognized by restriction enzymes have been identified in the *VDR* gene, including the *FokI* (*rs2228570*), *BsmI* (*rs1544410*), *ApaI* (*rs7975232*), and *TaqI* (*rs731236*), and their association with CD risk is still a matter of debate [8,9,10,11,12,13,14]. Ethnicity and the sample size of some of the studies are among the main reasons that explain the discrepancies reported. Of interest, in several cohorts of patients in which no association was found with CD risk, some of these polymorphisms were linked to more aggressive disease and a higher incidence of surgery [7,15,16]. In particular, the t allele in the *TaqI* polymorphism has been associated with reduced VDR protein levels in both epithelial cells and fibroblasts, which were increased by treatment with vitamin D [17].

Here, we aim to investigate the allelic and genotypic distributions of four common SNPs in the *VDR* gene, *FokI*, *BsmI*, *ApaI*, and *TaqI*, in a cohort of Caucasian CD patients and their association with clinical characteristics of the disease. Our data reveal a strong linkage disequilibrium between *TaqI* and *ApaI*, which in turn is associated with *BsmI*, and the results show that homozygosis for the a allele of the *ApaI* SNP is associated with a lower risk of both a penetrating behavior and perianal fistulas in CD patients and also with lower levels of fibrotic and inflammatory markers in primary intestinal fibroblasts.

## 2. Materials and Methods

### 2.1. Patients

In all cases, patients included in this study were recruited from the Gastroenterology Service of the Hospital of Manises (Valencia, Spain) following the Helsinki declaration recommendations. All patients were of European Caucasian ethnicity and had been followed up and treated in the hospital. This study was approved by the CEIM-Hospital Universitario y Politécnico la Fe (Valencia, Spain), with the protocol approval number 2021-284-1 (12 May 2021). Written informed consent was obtained from all patients.

Blood samples were obtained from a cohort of 115 CD patients (Table 1). We collected demographic and clinical data from them, including age, gender, age at diagnosis, family history, smoking status, disease location, disease behavior, presence of perianal fistulas, development of extraintestinal manifestations, and treatment history. In this study, we also included blood samples from non-IBD patients as control samples (55% female, 45% male). The mean ± SEM age of the controls was 39.8 ± 9.1 years, which was not significantly different than that of the CD patients (38.6 ± 1.2).

### 2.2. Primary Fibroblast Isolation

Primary intestinal fibroblasts were isolated from the surgical resections obtained from a second cohort of patients (Table 2), as previously reported [17,18]. In brief, the tissue was cut into small pieces and incubated with agitation in HBSS-EDTA for 30 min at 37 °C. After this step, a digestion of the pieces was performed with collagenase I (1 mg/mL), hyaluronidase (2 mg/mL), and DNAse (1 µL/mL) in PBS for 30 min at 37 °C. Finally, the explants were seeded in a Petri dish with the culture medium. The medium (DMEM high glucose, Sigma-Aldrich, Steinheim, Germany) was supplemented with FCS 20%, penicilin/streptomycin (100 µg/mL), gentamycin (100 µg/mL), amphotericin B (2 µg/mL), and ciprofloxacin (16 µg/mL). Fibroblasts from passages 6 to 8 were used.

### 2.3. DNA Extraction and Genotyping

Blood samples were genotyped for the four SNPs in the *VDR* gene, *ApaI* (*rs7975232*), *FokI* (*rs2228570*), *BsmI* (*rs1544410*), and *TaqI* (*rs731236*), while intestinal fibroblasts were only genotyped for the *ApaI* SNP. From both samples, genomic DNA was extracted from EDTA or citrated blood samples with the QIAamp^®^ DNA Mini kit (Qiagen, Hilden, Germany), following the manufacturer’s instructions. After extraction, DNA concentrations were measured by spectrophotometry with a NanoDrop^®^, ND1000 (Thermo Fisher Scientific, Waltham, MA, USA). Genotyping was carried out by polymerase chain reaction–restriction fragment length polymorphism (RFLP-PCR) [15,17]. DNA fragments containing the SNPs were amplified by PCR using 0.25 units of rTaq™ DNA Polymerase (Takara Bio Inc., Shiga, Japan) and the sense/antisense primers (Table 3). PCR products were restricted with the specific restriction enzyme (New England Biolabs, Ipswich, MA, USA) (Table 3) and resolved in 2% agarose gels to visualize the restriction fragments. The presence of restriction sites was coded with lower-case letters (*ApaI*: a; *BsmI*: b; *FokI*: f; and *TaqI*: t), and the absence of restriction sites with upper-case letters (*ApaI*: A; *BsmI*: B; *FokI*: F; and *TaqI*: T). We observed that the genotyped SNPs were in Hardy–Weinberg equilibrium in the CD patients and in the controls.

### 2.4. Real Time-PCR

Total RNA from the fibroblasts was isolated with an Illustra RNAspin Mini RNA isolation Kit (GE Healthcare Life Science, Amersham, UK), and 1 µg was used to obtain cDNA with the PrimeScript RT reagent Kit (Takara Bio Inc.). Real-time PCR was performed with the SYBR^®^ Premix Ex Taq (Takara Bio Inc.) in a LightCycler thermocycler (Roche Diagnostics, Penzberg, Germany). Specific oligonucleotides were designed according to the reported sequences and are shown in Table 4. β-actin was used as a housekeeping gene, and the results are expressed as the ΔCT between the CT gene and CT β-actin (CT: Threshold Cycle).

### 2.5. Statistical Analysis

For clinical data analysis, contingency tables considering the 3 genotypes of the specific SNP were analyzed by the χ^2^ test (3 × 2 tables). In some cases, we also compared them using Fisher’s exact test (2 × 2 tables), with one genotype vs. the other two of the specific SNP, as well as between haplotypes. Data obtained from cultured fibroblasts are presented as mean ± SEM, and normality tests were used to check the normal distribution of data. For the statistical analyses of gene expression between AA and aa genotypes in isolated fibroblasts, we used the *t*-test or Mann–Whitney test for independent samples, as appropriate. A *p* value < 0.05 was statistically significant.

## 3. Results

### 3.1. No Association Is Detected between CD Risk and the VDR FokI, BsmI, ApaI, and TaqI Gene Polymorphisms

To study a potential association between several SNPs in the *VDR* gene and CD risk, we genotyped a cohort of 115 CD patients and 20 healthy donors. The results showed that the genotypic frequencies of *VDR FokI*, *ApaI*, *BsmI*, and *TaqI* were not significantly different between the CD patients and healthy controls (Table 5).

### 3.2. A Linkage Disequilibrium between BsmI, ApaI, and TaqI Is Detected

Next, we proceeded to compare the presence of the different polymorphisms with each other to see if there were any possible relationships of inheritance in “clusters” or haplotypes (Table 6). The results reveal that the *FokI* SNP was not in linkage disequilibrium with any of the other three. In contrast, we detected a clear and significant association between the BB and AA genotypes and between these genotypes and the tt genotype (Table 6).

### 3.3. CD Patients Carrying the aa Genotype Have a Lower Risk of Developing a Perianal Fistula

**Figure 1:**
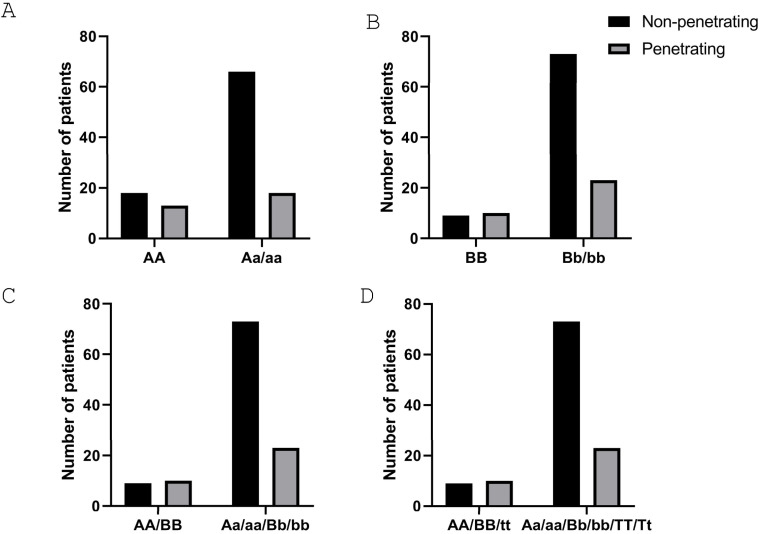
CD patients carrying the a, b, or T alleles in the *ApaI*, *BsmI*, and *TaqI* SNPs of the *VDR* gene, respectively, have a lower risk of a penetrating behavior. (**A**,**B**) Graphs show the association between the penetrating behavior in CD and the number of patients carrying the AA genotype (**A**) (*p* = 0.0347) and the BB genotype (**B**) (*p* = 0.0235). (**C**) Graph shows the association between the penetrating behavior in CD and the number of patients carrying the AA/BB haplotype (*p* = 0.0235). (**D**) Graph shows the association between the penetrating behavior in CD and the number of patients carrying the triple haplotype AA/BB/tt (*p* = 0.0235). The *p*-value corresponds to statistical analysis by the χ^2^ test of a contingency table for the two different conditions in each graph.

Next, we analyzed the association of genetic *VDR* polymorphisms with clinical characteristics of the disease. Our results demonstrate that patients carrying the aa genotype have a lower risk of presenting with perianal fistulas when compared with any of the other two genotypes (Table 7). Regarding the risk of suffering a penetrating behavior, we detected significant differences (*p* = 0.0347) when comparing the AA genotype vs. the combination of the other two genotypes (Figure 1A). No significant differences were detected for any of the other variables analyzed (Table 7).

### 3.4. CD Patients Carrying the bb Genotype Have a Lower Risk of Requiring Surgery

Regarding the VDR *BsmI* SNP, data show that patients carrying the bb genotype had a lower risk of surgery when compared with any of the other two genotypes (Table 8). In a similar manner to that obtained with the *ApaI* SNP, the risk of suffering a penetrating behavior was statistically significant (*p* = 0.0235) when comparing the BB genotype with the combination of the other two genotypes (Figure 1B). No significant differences were detected for any of the other variables analyzed (Table 8).

Finally, when we analyzed patients carrying the haplotype AA/BB vs. all the others (Aa/Bb/aa/bb), we detected a lower and significant risk (*p* = 0.0235) of suffering a penetrating behavior in the latter ones (Figure 1C). This was also observed when we compared patients carrying the triple haplotype AA/BB/tt vs. all the other ones (*p* = 0.0235) (Figure 1D).

### 3.5. No Differences Were Detected in Any of the Clinical Parameters among the Three Genotypes in the FokI SNP of the VDR Gene

As shown in Table 9, the *FokI* genotypes in the *VDR* gene were not significantly associated with any clinical characteristic of the disease.

### 3.6. Fibroblasts Carrying the aa Genotype Expressed Lower Levels of Fibrotic Markers

**Figure 2:**
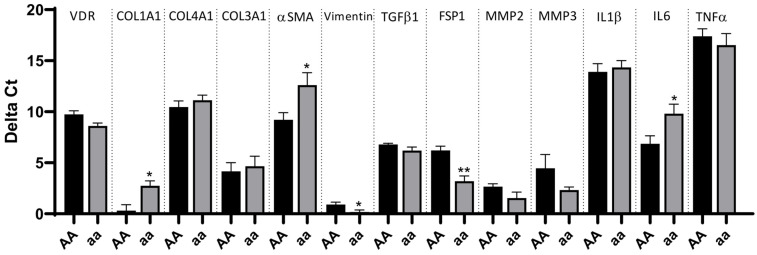
Increased expression of pro-inflammatory and profibrotic genes in intestinal fibroblasts from CD patients carrying the AA genotype. Intestinal fibroblasts were obtained from ileal surgical resections from CD patients homozygous for alleles A and a in the *ApaI* SNP of the *VDR* gene. Graphs show the delta Ct (Ct gene–Ct actin) of several cytokines, fibrotic genes, and *VDR* in fibroblasts homozygous for the a allele vs. the A allele. Bars represent mean ± S.E.M. (n = 5 for AA and n = 5 for aa). * *p* < 0.05 or ** *p* < 0.01 vs. the AA genotype in each specific gene by Student’s *t*-test or the Mann–Whitney test, as appropriate.

Fibroblasts were isolated from resections of CD patients and classified according to the genotype for the *ApaI* SNP. The analyses of the mRNA expression of different cellular markers in these cells showed remarkable differences. As shown in Figure 2, the mRNA expression of *COL1A1*, *αSMA* (involved in the contractile apparatus), and *IL6* was significantly higher in fibroblasts carrying the AA genotype than in those carrying the aa genotype. In contrast, the former exhibited significantly lower mRNA levels of both *VIMENTIN* and *FSP1* than the latter ones. A non-significant reduction in the mRNA expression of *VDR* was detected and associated with the AA genotype (Figure 2).

## 4. Discussion

Our results reveal an association between patients carrying the a allele of *ApaI* SNP in the *VDR* gene and a lower risk of suffering both a penetrating behavior and perianal fistula in CD. Isolated intestinal fibroblasts from CD patients carrying the aa genotype exhibited a lower gene expression of fibrotic markers, such as *COL1A1*, α-*SMA*, and *IL6*, and a higher expression of *FSP1*.

Our results in a cohort of Caucasian patients reveal that the genotypic frequencies of the four *VDR* SNPs analyzed, *FokI*, *BsmI*, *ApaI*, and *TaqI*, did not significantly differ between controls and CD patients, which suggests that the *VDR* polymorphisms are not associated with a higher CD risk. Controversy has been reported regarding this association, and the sample size and ethnicity have been argued as the reasons for these discrepancies [8,9,11,12]. We agree that the sample sizes of the present and previous studies constitute a limitation to answer this query, but it is important to note that the *VDR* rare allele frequencies obtained in our cohort of patients (*FokI* = 57.5%, *BsmI* = 44.5%, *ApaI* = 58%, and *TaqI* = 39.5%) were comparable to other studies, some of which included a larger sample size of the Caucasian population [19]. Our data also reveal that *FokI* is not associated with any other SNP, while *ApaI* is strongly associated with *BsmI*, and both exhibited a linkage disequilibrium with the *TaqI* SNP, as previously reported [20]. In line with this, we have detected an association between the risk of penetrating behavior and the genotypes AA and BB, which reinforces the reported association of this behavior with the tt genotype in the same cohort of patients [15]. The association of these genotypes with penetrating behavior in CD is further demonstrated in patients carrying the haplotype AA/BB/tt in the *VDR* gene, a haplotype that has also been associated with other diseases, such as depressive disorders [20]. Of interest, our data extend these observations and show for the first time that the aa genotype reduces the risk of perianal fistulas. This observation seems to be independent of the association found between the AA genotype with the penetrating behavior since, in line with previous studies [2], the percentage of patients with perianal fistulas in our cohort is similar among CD behaviors (36% for penetrating, 40% for stenotic, and 31% for inflammatory). Furthermore, our data reveal that patients carrying the BB genotype did not exhibit that risk, but they were associated with a higher surgery risk. Further studies with a larger population are required to better understand the association of the *ApaI*, *BsmI*, and *TaqI* SNPs with two characteristics of the disease, perianal fistulas and surgery, which are intimately related.

The etiopathogenic mechanisms of perianal fistulas are poorly understood, but matrix remodelling, the epithelial–mesenchymal transition, and a high inflammatory burden seem to be involved [21]. Fibroblasts not only play an essential role in matrix deposition but also as a source of cytokines involved in the perpetuation of inflammation [22,23]. Our data reveal in fibroblasts isolated from intestinal resections of CD patients a higher mRNA expression of a profibrotic gene, *αSMA* in cells carrying the AA genotype than in those carrying the aa genotype. αSMA is a molecule associated with the differentiation of fibroblasts to myofibroblasts [24], a process that is crucial for ECM deposition, which agrees with the higher mRNA expression of *COL1A1* also detected in these cells. Considering the anti-inflammatory and antifibrotic effects attributed to *VDR* [6] and the linkage disequilibrium between *ApaI* and *TaqI* SNPs, this observation may be related to the reduced VDR protein levels previously reported in patients carrying the tt genotype in the *TaqI* SNP [17]. Of interest, our data also show increased expression of *IL6* in fibroblasts carrying the AA genotype, suggesting higher levels of inflammatory cytokines associated with this variant, which not only matches with the higher *TNFα* levels detected in the serum of healthy children carrying this genotype [25] but also with the increased expression of *IL1β* previously detected in PBMC carrying the tt genotype [15]. Finally, fibroblasts homozygous for the A allele of *ApaI* exhibited lower levels of the apoptosis inducing factor, *FSP1*, than those carrying the aa genotype. The fact that fibroblasts analyzed in the present study were obtained from the intestines of CD patients with complications, suggests that a higher viability of these cells may be involved in CD complications [26].

## 5. Conclusions

In conclusion, our data reveal an association between CD patients carrying the a allele of *ApaI* SNP in the *VDR* gene and both a lower risk of perianal fistulas and a penetrating behavior, as well as with a reduced expression of fibrotic and inflammatory markers in intestinal fibroblasts. These findings suggest that the characterization of *VDR* polymorphisms may help clinicians better predict the risk of CD complications and optimize treatment, including VD supplementation, which has been shown to restore the reduced VDR protein levels detected in fibroblasts from CD patients [27], including those carrying the *TaqI* polymorphism [17].

## Figures and Tables

**Table 1:** Clinical characteristics of Crohn’s disease patients from whom blood was obtained.

CD Patients	n = 115
Gender	
Female	63 (54.7%)
Male	52 (45.2%)
Age at diagnosis (mean ± SD; years)	28.07 ± 12.67
A1 (<17 years)	15 (13.0%)
A2 (17–40 years)	78 (67.8%)
A3 (>40 years)	22 (19.1%)
Follow-up time (mean ± SD; years)	11.26 ± 10.62
Disease location	
L1—ileal	57 (49.6%)
L2—colonic	15 (13.0%)
L3—ileocolonic	33 (28.7%)
L4—upper GI ^a^	10 (7%)
Disease behavior	
B1—inflammatory	52 (45.2%)
B2—stricturing	32 (27%)
B3—penetrating	31 (26.9%)
Surgery	47 (40.8%)
Perianal fistula	41 (35.6%)
EIM ^b^	28 (24.3%)
Family history	11 (9%)
Smoker or ex-smoker	45 (38%)

^a^ Upper GI is always affected in addition to another site (L1, L2, or L3). ^b^ Extraintestinal manifestations.

**Table 2:** Clinical characteristics of CD patients from whom fibroblasts were obtained.

CD Patients	n = 10
Gender	
Female	7 (70%)
Male	3 (30%)
Age (mean ± SD; years)	47.9 ± 20.32
Disease location	
L1—ileal	7 (70%)
L2—colonic	0 (0%)
L3—ileocolonic	3 (30%)
L4—upper GI ^a^	0 (0%)
Disease behavior	
B1—inflammatory	0 (0%)
B2—stricturing	5 (50%)
B3—penetrating	5 (50%)

^a^ Upper GI is always affected in addition to another site (L1, L2, or L3).

**Table 3:** Primers and restriction enzymes used.

	Sense Primer (5′–3′)	Antisense Primer (5′–3′)	*T* Annealing (°C)	Enzyme Restriction	*T* Enzyme (°C)
*rs7975232 (ApaI)*	CAGAGCATGGACAGGGAGCAA	GCAACTCCTCATGGCTGAGGTCTC	60	*ApaI* (R0114S)	25
*rs1544410 (BsmI)*	CAACCAAGACTACAAGTACCGCGTCAGTGA	AACCAGCGGGAAGAGGTCAAGGG	65	*BsmI* (R0134S)	65
*rs2228570 (FokI)*	AGCTGGCCCTGGCACTGACTCTGCTCT	ATGGAAACACCTTGCTTCTTCCCTCTC	60	*FokI* (R0109S)	37
*rs731236 (TaqI)*	ATGCACGGAGAAGTCACTGG	GGTCGGCTAGCTTCTGGATC	60	*TaqI* (R0149S)	65

*T*: temperature.

**Table 4:** Sequences of primers used in real time-PCR.

	Sense Primer (5′–3′)	Antisense Primer (5′–3′)
*VDR*	TGGAGACTTTGACCGGAACG	AAGGGGCAGGTGAATAGTGC
*COL1A1*	GGAGCAGACGGGAGTTTCTC	CCGTTCTGTACGCAGGTGAT
*COL4A1*	CCGGATCACATTGACATGAAACC	CCGGATCACATTGACATGAAACC
*COL3A1*	CGCCCTCCTAATGGTCAAGG	TTCTGAGGACCAGTAGGGCA
*ACTA2 (αSMA)*	GACCTTTGGCTTGGCTTGTC	AGCTGCTTCACAGGATTCCC
*Vimentin*	ATGAAGGAGGAAATGGCTCGTC	GGGTATCAACCAGAGGGAGTGAA
*TNFα*	GCTGCACTTTGGAGTGATCG	GGGTTTGCTACAACATGGGC
*TGFβ1*	GGGCTACCATGCCAACTTCT	GACACAGAGATCCGCAGTCC
*FSP1*	TCTTGGTTTGATCCTGACTGCT	CCTGTTGCTGTCCAAGTTGC
*MMP2*	CATTCCCTGCAAAGAACACA	GTATTTGATGGCATCGCTCA
*MMP3*	TCCTACTGTTGCTGTGCGTG	CTTCCCCGTCACCTCCAATC
*IL1β*	GCTCGCCAGTGAAATGATGG	TCGTGCACATAAGCCTCGTT
*IL6*	ATGAGGAGACTTGCCTGGTG	CTGGCATTTGTGGTTGGGTC
*ACTB (βActin)*	GGACTTCGAGCAAGAGATGG	AGCACTGTGTTGGCGTACAG

**Table 5:** Genotype frequencies of the vitamin D receptor variants in the control and CD patients.

		Control	CD	Total	*p*-Value
*FokI*	FF	7 (35%)	45 (39.1%)	52	0.6356
	Ff	9 (45%)	56 (48.7%)	65	
	**ff**	4 (20%)	14 (12.2%)	18	
*ApaI*	AA	8 (40%)	31 (26.9%)	39	0.1848
	Aa	5 (25%)	54 (46.9%)	59	
	**aa**	7 (35%)	30 (26.1%)	37	
*Bsml*	BB	6 (30%)	19 (16.5%)	25	0.2858
	Bb	6 (30%)	51 (44.3%)	57	
	**bb**	8 (40%)	45 (39.2%)	53	
*TaqI*	**TT**	8 (40%)	45 (39.1%)	53	0.3981
	Tt	6 (30%)	49 (42.6%)	55	
	tt	6 (30%)	21 (18.2%)	27	

The *p*-value corresponds to the statistical analysis by the χ^2^ test of a contingency table for the three genotypes. In bold: reference genotypes.

**Table 6:** Distribution of CD patients according to the different genotypes for each SNP in the *VDR* gene.

SNP		*FokI*			*BsmI*			*ApaI*			*TaqI*		
		FF	Ff	ff	BB	Bb	bb	AA	Aa	aa	TT	Tt	tt
***FokI***	FF				6	20	19	9	25	11	20	19	6
	Ff				10	26	20	19	22	15	19	25	12
	ff				3	5	6	3	7	4	6	5	3
***p***					0.8182			0.4772			0.7437		
***BsmI***	BB							19	0	0	0	0	19
	Bb							11	40	0	3	47	1
	bb							1	14	30	42	2	1
***p***								**<0.001**			**<0.001**		
***ApaI***	AA										2	9	20
	Aa										14	39	1
	aa										29	1	0
***p***											**<0.001**		

The *p*-value corresponds to statistical analysis by the χ^2^ test of a contingency table. In bold, analyses with *p* < 0.05.

**Table 7:** Classification of Crohn’s disease patients by genotypes in the *ApaI* SNP on the *VDR* gene.

Variable		AA (n = 31)	Aa (n = 54)	aa (n = 30)	*p*-Value ^a^
Gender					
	Female	18	29	16	0.9114
	Male	13	25	14	
Age at onset					
	A1 + A2	28	41	24	0.2647
	A3	3	13	6	
Location					
	L1	17	22	18	0.3153
	L2	3	11	1	
	L3	9	15	9	
	L4	2	6	2	
Behavior					
	B1	13	25	14	0.2098
	B2	5	18	9	
	B3	13	11	7	
Perianal fistula					
	Yes	16	19	6	**0.0360**
	No	15	35	24	
Surgery					
	Yes	16	20	11	0.3628
	No	15	34	19	
EIM ^b^					
	Yes	5	15	8	0.4563
	No	26	39	22	

^a^ The *p*-value corresponds to statistical analysis by the χ^2^ test of a contingency table for the 3 genotypes and 2, 3, or 4 related conditions. ^b^ Extraintestinal manifestations.

**Table 8:** Classification of Crohn’s disease patients by genotypes in the *BsmI* SNP on the *VDR* gene.

Variable		BB (n = 19)	Bb (n = 51)	bb (n = 45)	*p*-Value ^a^
Gender					
	Female	12	28	24	0.5434
	Male	7	23	21	
Age at onset					
	A1 + A2	17	42	35	0.5359
	A3	2	9	10	
Location					
	L1	10	25	22	0.8812
	L2	3	6	4	
	L3	4	14	16	
	L4	2	6	3	
Behavior					
	B1	6	25	18	0.1036
	B2	3	16	14	
	B3	10	10	13	
Perianal fistula					
	Yes	7	22	13	0.3509
	No	12	29	32	
Surgery					
	Yes	13	14	20	**0.0067**
	No	6	37	25	
EIM ^b^					
	Yes	2	13	13	0.2850
	No	17	38	32	

^a^ The *p*-value corresponds to statistical analysis by the χ^2^ test of a contingency table for the 3 genotypes and 2, 3, or 4 related conditions. In bold, analysis with *p* < 0.05. ^b^ Extraintestinal manifestations.

**Table 9:** Classification of Crohn’s disease patients by genotypes in the *FokI* SNP on the *VDR* gene.

Variable		FF (n = 45)	Ff (n = 56)	ff (n = 14)	*p*-Value ^a^
Gender					
	Female	25	33	6	0.5564
	Male	20	23	8	
Age at onset					
	A1 + A2	40	43	10	0.1939
	A3	5	13	4	
Location					
	L1	21	30	7	0.4100
	L2	8	4	3	
	L3	12	19	2	
	L4	4	3	2	
Behavior					
	B1	20	26	5	0.8227
	B2	14	13	5	
	B3	11	17	4	
Perianal fistula					
	Yes	17	17	6	0.5875
	No	28	39	8	
Surgery					
	Yes	20	23	4	0.5726
	No	25	33	10	
EIM ^b^					
	Yes	10	15	3	0.8370
	No	35	41	11	

^a^ The *p*-value corresponds to statistical analysis by the χ^2^ test of a contingency table for the 3 genotypes and 2, 3, or 4 related conditions. In bold, analysis with *p* < 0.05. ^b^ Extraintestinal manifestations.

## Data Availability

The data used in this manuscript are not publicly available because of participants’ privacy concerns but are available upon reasonable request.

## References

[1] Verstockt B., Bressler B., Martinez-Lozano H., McGovern D., Silverberg M.S. (2022). Time to Revisit Disease Classification in Inflammatory Bowel Disease: Is the Current Classification of Inflammatory Bowel Disease Good Enough for Optimal Clinical Management?. Gastroenterology.

[2] Householder S., Picoraro J.A. (2022). Diagnosis and Classification of Fistula from Inflammatory Bowel Disease and Inflammatory Bowel Disease-Related Surgery. Gastrointest. Endosc. Clin. N. Am..

[3] Geldof J., Iqbal N., LeBlanc J.F., Anandabaskaran S., Sawyer R., Buskens C., Bemelman W., Gecse K., Lundby L., Lightner A.L. (2022). Classifying perianal fistulising Crohn’s disease: An expert consensus to guide decision-making in daily practice and clinical trials. Lancet Gastroenterol. Hepatol..

[4] Bernstein C.N., Panaccione R., Nugent Z., Marshall D.A., Kaplan G.G., Vanner S., Dieleman L.A., Graff L.A., Otley A., Jones J. (2024). Crohn’s Disease Phenotypes and Associations with Comorbidities, Surgery Risk, Medications and Nonmedication Approaches: The MAGIC in IMAGINE Study. Inflamm. Bowel Dis..

[5] Annese V. (2020). Genetics and epigenetics of IBD. Pharmacol. Res..

[6] Bakke D., Sun J. (2018). Ancient Nuclear Receptor VDR with New Functions: Microbiome and Inflammation. Inflamm. Bowel Dis..

[7] Martin K., Radlmayr M., Borchers R., Heinzlmann M., Folwaczny C. (2002). Candidate genes colocalized to linkage regions in inflammatory bowel disease. Digestion.

[8] Simmons J.D., Mullighan C., Welsh K.I., Jewell D.P. (2000). Vitamin D receptor gene polymorphism: Association with Crohn’s disease susceptibility. Gut.

[9] Xue L.N., Xu K.Q., Zhang W., Wang Q., Wu J., Wang X.Y. (2013). Associations between vitamin D receptor polymorphisms and susceptibility to ulcerative colitis and Crohn’s disease: A meta-analysis. Inflamm. Bowel Dis..

[10] Wang L., Wang Z.T., Hu J.J., Fan R., Zhou J., Zhong J. (2014). Polymorphisms of the vitamin D receptor gene and the risk of inflammatory bowel disease: A meta-analysis. Genet. Mol. Res..

[11] Xia S.L., Lin X.X., Guo M.D., Zhang D.G., Zheng S.Z., Jiang L.J., Jin J., Lin X.Q., Ding R., Jiang Y. (2016). Association of vitamin D receptor gene polymorphisms and serum 25-hydroxyvitamin D levels with Crohn’s disease in Chinese patients. J. Gastroenterol. Hepatol..

[12] Naderi N., Farnood A., Habibi M., Derakhshan F., Balaii H., Motahari Z., Agah M.R., Firouzi F., Rad M.G., Aghazadeh R. (2008). Association of vitamin D receptor gene polymorphisms in Iranian patients with inflammatory bowel disease. J. Gastroenterol. Hepatol..

[13] Luo Y.Y., Shu X.L., Zhao H., Yu J.D., Ma M., Chen J. (2013). Association between vitamin D receptor gene polymorphisms and pediatric Crohn’s disease in China: A study based on gene sequencing. Zhongguo Dang Dai Er Ke Za Zhi.

[14] Limketkai B.N., Singla M.B., Rodriguez B., Veerappan G.R., Betteridge J.D., Ramos M.A., Hutfless S.M., Brant S.R. (2020). Levels of Vitamin D Are Low After Crohn’s Disease Is Established but Not Before. Clin. Gastroenterol. Hepatol..

[15] Gisbert-Ferrandiz L., Salvador P., Ortiz-Masia D., Macias-Ceja D.C., Orden S., Esplugues J.V., Calatayud S., Hinojosa J., Barrachina M.D., Hernandez C. (2018). A Single Nucleotide Polymorphism in the Vitamin D Receptor Gene Is Associated with Decreased Levels of the Protein and a Penetrating Pattern in Crohn’s Disease. Inflamm. Bowel Dis..

[16] Cusato J., Cafasso C., Antonucci M., Palermiti A., Manca A., Caviglia G.P., Vernero M., Armandi A., Saracco G.M., D’Avolio A. (2024). Correlation between Polymorphisms of Vitamin D Metabolism Genes and Perianal Disease in Crohn’s Disease. Biomedicines.

[17] Gisbert-Ferrandiz L., Cosin-Roger J., Hernandez C., Macias-Ceja D.C., Ortiz-Masia D., Salvador P., Wildenberg M.E., Esplugues J.V., Alos R., Navarro F. (2020). The vitamin D receptor Taq I polymorphism is associated with reduced VDR and increased PDIA3 protein levels in human intestinal fibroblasts. J. Steroid Biochem. Mol. Biol..

[18] Macias-Ceja D.C., Ortiz-Masia D., Salvador P., Gisbert-Ferrandiz L., Hernandez C., Hausmann M., Rogler G., Esplugues J.V., Hinojosa J., Alos R. (2019). Succinate receptor mediates intestinal inflammation and fibrosis. Mucosal Immunol..

[19] Hughes D.J., McManus R., Neary P., O’Morain C., O’Sullivan M. (2011). Common variation in the vitamin D receptor gene and risk of inflammatory bowel disease in an Irish case-control study. Eur. J. Gastroenterol. Hepatol..

[20] Lye M.S., Tor Y.S., Tey Y.Y., Shahabudin A., Loh S.P., Ibrahim N., Stanslas J., Rosli R., Ling K.H. (2021). *BsmI*-*ApaI*-*TaqI* TAC (BAt) Haplotype of Vitamin D Receptor Gene Is Associated with Increased Risk of Major Depressive Disorder. J. Mol. Neurosci..

[21] Cao S., Colonna M., Deepak P. (2023). Pathogenesis of Perianal Fistulising Crohn’s Disease: Current Knowledge, Gaps in Understanding, and Future Research Directions. J. Crohn’s Colitis.

[22] Lenti M.V., Santacroce G., Broglio G., Rossi C.M., Di Sabatino A. (2024). Recent advances in intestinal fibrosis. Mol. Asp. Med..

[23] Yang W., Yu T., Cong Y. (2024). Stromal Cell Regulation of Intestinal Inflammatory Fibrosis. Cell. Mol. Gastroenterol. Hepatol..

[24] Zhang H., Zhou Y., Wen D., Wang J. (2023). Noncoding RNAs: Master Regulator of Fibroblast to Myofibroblast Transition in Fibrosis. Int. J. Mol. Sci..

[25] Ferrer-Suay S., Alonso-Iglesias E., Tortajada-Girbes M., Carrasco-Luna J., Codoner-Franch P. (2021). Vitamin D receptor gene *ApaI* and *FokI* polymorphisms and its association with inflammation and oxidative stress in vitamin D sufficient Caucasian Spanish children. Transl. Pediatr..

[26] Seco-Cervera M., Ortiz-Masia D., Macias-Ceja D.C., Coll S., Gisbert-Ferrandiz L., Cosin-Roger J., Bauset C., Ortega M., Heras-Moran B., Navarro-Vicente F. (2024). Resistance to apoptosis in complicated Crohn’s disease: Relevance in ileal fibrosis. Biochim. Biophys. Acta Mol. Basis Dis..

[27] Gisbert-Ferrándiz L., Cosín-Roger J., Hernández C., Macias-Ceja D.C., Ortiz-Masiá D., Salvador P., Esplugues J.V., Hinojosa J., Navarro F., Calatayud S. (2020). Diminished Vitamin D Receptor Protein Levels in Crohn’s Disease Fibroblasts: Effects of Vitamin D. Nutrients.

